# Clerkship Students’ Use of Clinical Reasoning Concepts After a Pre-clinical Reasoning Course

**DOI:** 10.1007/s11606-024-09279-4

**Published:** 2025-01-02

**Authors:** Shradha A. Kulkarni, Gurpreet Dhaliwal, Arianne Teherani, Denise M. Connor

**Affiliations:** 1 Department of Medicine, Baylor College of Medicine, Houston, USA; 2 Department of Medicine, University of California San Francisco, San Francisco, USA; 3 Medical Service, San Francisco VA Medical Center, San Francisco, USA

**Keywords:** clinical reasoning, undergraduate medical education, diagnosis, clinical learning environment, clinical clerkships

## Abstract

**Background:**

Many medical schools have incorporated clinical reasoning (CR) courses into their pre-clinical curricula to address the quality and safety issue of diagnostic error. It is unknown how students use concepts and practices from pre-clinical CR courses once in clerkships.

**Objective:**

We sought to understand how students utilize CR concepts from a pre-clinical course during clerkships and to identify facilitators and barriers to the use of reasoning concepts.

**Design:**

We used structured interviews to gain insight into medical students’ experiences with CR concepts in clerkships.

**Participants:**

We interviewed 16 students who had completed a pre-clinical CR course and subsequently completed a neurology, internal medicine, or pediatrics clerkship.

**Approach:**

We used constructivist grounded theory to perform a qualitative analysis and to develop a theoretical model to describe findings.

**Key Results:**

Insights fell into three main areas: (1) CR concept carryover, representing concepts taught in the CR course, such as problem representation, illness scripts, schema, and prioritized differential diagnosis, which were utilized in clerkships; (2) CR concept reinforcers, which included the clerkship setting and supervising physicians who emphasized and provided feedback on CR; and (3) CR concept diminishers, which included time constraints and supervisors who were unfamiliar with or did not reinforce CR concepts.

**Conclusions:**

Concepts taught in a pre-clinical CR course influenced how students prepared for and navigated clinical encounters. Contextual factors both enhanced and inhibited the utilization of CR concepts. Our findings align with social learning theories including social cognitive theory and ecological psychology. This contextual view—taking into account interactions between personal, social, and environmental factors—can help educators integrate CR education from the classroom to the clinical setting.

**Supplementary Information:**

The online version contains supplementary material available at 10.1007/s11606-024-09279-4.

## INTRODUCTION

For the past decade, diagnostic error has been identified as a quality and safety issue, and the importance of teaching clinical reasoning (CR) skills from the beginning of medical training has been emphasized by educators.^1^ Consensus statements have called for longitudinal CR curricula integrated throughout undergraduate medical training.^2^ Medical schools have implemented pre-clinical CR courses using a range of pedagogical approaches.^3–10^ However, implementation of CR curricula during the clinical phase of medical school has been limited. A majority of U.S. internal medicine clerkship directors support a program of CR instruction throughout the 4 years of medical school,^11^ but few clerkships offer sessions devoted to CR,^12^ citing curricular time and faculty expertise as barriers.

To create effective longitudinal CR instruction across the 4 years of medical school, educators need to understand which reasoning concepts and skills from pre-clinical courses are useful when learners reach the clinical setting and what factors impact their use. The challenges students face in making connections between the classroom and the clinical workplace have been examined for basic science courses like biochemistry and anatomy.^13,14^ However, there are no studies on how the CR concepts and practices taught in the classroom are utilized, reinforced, or diminished in the clerkship environment. Insight into how clerkship students use knowledge from CR courses would help educators align pre-clinical learning with the clinical setting, focus faculty development efforts to ensure clinical teachers are able to reinforce reasoning concepts and skills, and create opportunities to extend key reasoning concepts in clerkships.

In this study, we sought to understand how CR concepts taught in a pre-clinical course were used in the clinical environment and to identify facilitators and barriers to their use in the clerkship environment. We hoped to gain insights that would inform future CR curricular design.

## METHODS

### Setting

In 2018, we launched a pre-clinical reasoning course at the University of California, San Francisco (UCSF) School of Medicine.^15^ Many frameworks for understanding CR have been described, with some scholars emphasizing knowledge structures, while others focus on cognitive processes. We find both aspects of reasoning relevant and integrated aspects of both into our curricular design. The CR course emphasizes knowledge architecture (e.g., illness scripts and diagnostic schema), provides case-based opportunities to practice reasoning skills like developing problem representations and articulating prioritized differential diagnoses, and invites consideration of other domains (e.g., communication, interprofessional collaboration, bias) that impact reasoning.^15,16^ Reasoning concepts are introduced periodically during the first 18 months of medical school and consolidated in an immersive 3-week CR course before the start of clerkships using a combination of lectures, panels, and interactive small groups (see Appendix 1 for details). The latter are led by faculty in internal medicine, pediatrics, neurology, emergency medicine, and family medicine. The goals of the course are to teach medical students how to develop (1) a structured approach to analyzing and discussing a diagnostic problem and (2) a comprehensive understanding of the domains that impact the diagnostic reasoning process and influence the risk for diagnostic error. The CR course emphasizes diagnostic rather than management reasoning because diagnosis is foundational to clinical practice, aligns with pathophysiology and disease-oriented content taught during the pre-clerkship curriculum, and precedes management in common assessment scales (e.g., RIME^17^).

### Participants

We invited all students who completed clerkships in medicine, neurology, or pediatrics during the first half of their clerkship year to participate. Medicine, neurology, and pediatrics clerkships were chosen as inclusion criteria given their emphasis on diagnosis. However, students were able to reflect on diagnosis-focused experiences from any of their rotations during interviews. No students had completed their year-long, longitudinal family medicine clerkship nor the fourth-year emergency medicine clerkship; therefore, these diagnostically focused rotations were not part of our inclusion criteria. All students had completed the CR course before clerkships. Invitations were sent by email with several reminder emails sent during the first few months of clerkships. In person reminders were shared during clerkship didactic sessions. Participants received a $20 gift card.

### Research Approach

We opted for a qualitative approach as this methodology was well suited for an exploratory study of a less well-understood phenomenon, and enabled us to delve into students’ experiences with and perceptions of reasoning in the clerkships. As there was no pre-existing theory specific to the CR skills of learners transitioning from the classroom to the clerkships, we selected constructivist grounded theory to analyze qualitative data and develop a theoretical model to illustrate our findings.^18–20^ A grounded theory approach provided an opportunity to develop a deeper understanding of students’ metacognitive approaches to and influences on their reasoning. A constructivist approach enabled us to use our own understanding of reasoning to interpret and analyze the data, and to identify themes and perspectives that might otherwise have been unnoticed. Our constructivist research paradigm also afforded exploration of CR as a complex process influenced by individuals’ unique learning experiences in different contexts.^20^ The study received exempt status from the UCSF IRB.

### Instrument and Interviews

Two investigators (DC and GD) designed a structured interview guide to explore how students thought through and communicated about the diagnostic process in clerkships. Questions were revised with input from medical education faculty: one non-clinical medical education scientist, two physician leaders of a longitudinal clinical skills course, and an interprofessional education scholarship group, who recommended that we consider sensitizing concepts as we revised our interview guide. The interview guide was pilot tested with two clerkship students and adjusted for clarity. Two senior medical students conducted interviews to reduce the power differential between participants and interviewers with the aims of enhancing participants’ openness to sharing experiences, reducing bias that could be introduced if faculty invested in the curriculum were to interject leading questions, and decreasing the potential for influencing participants’ responses. Student interviewers were trained to follow the interview guide and avoid adding prompting questions outside of general extenders such as “tell me more.” The interview guide (including notes about sensitizing concepts) is provided in Appendix 2.

To prime their memory of a specific example of a diagnostically challenging case, participants were asked to review an admission note from a recent clerkship, but interviews were not limited to that patient encounter. The interviewer asked students to describe their thought processes before, during, and after seeing a patient, and how they shared their reasoning with others. Students were also asked to reflect on the clinical learning environment (including interactions with other team members such as supervisors) and how it influenced their reasoning. Finally, students were asked to describe clinical situations where they noted a risk for diagnostic error.

### Data Collection

Interviewers received training in qualitative interviewing from an education scientist (AT). Interviews were conducted by phone in the spring of 2019 and 2020, lasted 30–60 min, and were recorded and transcribed. Recordings were de-identified and stored on a secure research drive. Only general data about participants (clerkship rotations and clinical sites) were retained.

### Analysis

We used a constant comparative, iterative process to analyze interview data and develop a codebook. Each investigator (DC, GD, SK, AT) independently coded several transcripts and met to discuss codes and create a codebook. Three investigators (DC, GD, and SK) then further refined the codebook. Two investigators then independently and sequentially coded each transcript, and one investigator (DC) compared the two independent codes. These three investigators met to resolve any differences in coding and to decide whether any new codes should be added. After ten transcripts were coded, no new major experiences or perspectives appeared in the data.^19^

All transcripts were coded and organized using Dedoose analytic software (Sociocultural Research Consultants, LLC, Manhattan Beach, California). Three investigators then utilized the coding scheme to undertake an in-depth analytic process, dividing the major code categories between them and reviewing all quotes for each code, identifying over-arching themes and concepts, and looking for connections between codes. The investigators developed a model that described the major themes and relationships between themes, and noted connections between themes in the data and concepts taught in the pre-clinical CR course.

### Reflexivity

Our research team included two clinician-educators (DC, GD) with experience in CR curriculum design and teaching, one clinician-educator (SK) with experience as an instructor in the CR course, and one education scientist (AT) with experience in qualitative methodology. DC was the main curriculum designer and director of UCSF’s pre-clinical CR course during the study.

DC emphasized the situated, contextual nature of reasoning; GD brought a cognitivist lens to the reasoning process; SK provided the perspective of an early-career clinician educator who recently encountered concepts of CR throughout medical school and residency; AT offered the perspective of a non-clinician scholar with a focus on learning theories*.* Our team believes that both knowledge architecture and cognitive processes are relevant to reasoning, as opposed to considering these to be mutually exclusive frameworks, and brought an integrated perspective to our analysis.

## RESULTS

We interviewed 16 clerkship students; students shared diverse diagnostic scenarios in their interviews (e.g., cough, dizziness, weight loss). Our findings were grouped into three major themes: (1) CR concept carryover—reasoning concepts, terms, and practices taught in the CR course that were used by students in clerkships; (2) CR concept reinforcers—factors in the clerkship environment that reinforced or extended application of reasoning concepts and skills; and (3) CR concept diminishers—factors that limited application.

### Theme 1: CR Concept Carryover

Clerkship students identified and used many of the concepts and approaches taught in the CR course including problem representation, illness scripts, schema, and prioritized differential diagnoses (collectively referred to as the CR framework). Students frequently cited the utility of diagnostic schema:

*“I honestly used a lot of the schemas that I came up with in the [pre-clinical CR course], or ones that I had heard on Clinical Problem Solvers [podcast and website*^21^*]… I just kind of mapped it out, mapped things out on a paper, and then try and think about, like, ‘Does this fit or not? Why not?’”*

*“…there are many instances…in Peds now when I have been able to think [about] things in big buckets and it’s very clear this is how clinicians actually are able [to] be like ‘Oh, what things could fall under inflammatory or what things could fall under malignancies or infectious?’”*

*“I would say that a schema for me is more of a starting place when I don’t have a good idea of what the patient’s coming in with. When you just have a word or a symptom to point to. It’s cough, or it’s ‘I lost consciousness,’ or ‘I have abdominal pain.’ Then the schema is a way to get from square one to move along the path to narrow down your diagnosis.”*

Students reported approaching the history and exam in a hypothesis-driven manner, another concept emphasized in the CR course.

*“…And then thinking about what signs and symptoms I’m going to be looking for on a physical exam and what kind of history questions I’m going to be asking to further differentiate organ system and systemic kinds of things.”*

Students described prioritizing and justifying their differential diagnosis, and intentionally including “can’t miss” diagnostic considerations—all skills which were emphasized in the CR course.

*“For other things you have to kind of think about how serious is this diagnosis? What would be the cost if I missed it? Then what is on my pros and cons list of this diagnosis? Like how many things match with this, what I have in my mind [about] this diagnosis versus what doesn’t fit, and kind of balancing those two things is something… Cancer is a very serious diagnosis, but…it’s rarely like a hospital emergency, so that’s not necessarily something that you would put as your ‘can’t miss’ diagnosis.”*

The CR course highlighted the importance of partnering with patients and families. Students expressed understanding of how this partnership provided information that influenced diagnosis.

“*I hadn’t thought of the fact that the ARVs [antiretrovirals] might be causing the diarrhea, and that was in part led by the patient. He had said, oh, well, my partner. He had these ARVs, and he had diarrhea, so maybe it’s that. I had picked that up from the patient.”*

Echoing discussions about the importance of interprofessional communication on the diagnostic process in the CR course, students noted that input from interprofessional colleagues could influence their reasoning process or modify their diagnostic conclusions.

*“I usually like to check in with the nurse and just make sure if the patient has already given the nurse a boiled down version of what’s happening.”*

*“I had told the nurses right away, like, ‘He has heartburn, but I think it might be cardiac,’ and they kind of dismissed my concern, so I didn't bring it up to my attending until after morning report.”*

Cognitive biases, the tendencies of the brain to interpret and filter information based on prior experiences and assumptions,^22^ were discussed as a source of error during the CR course. Students reported how anchoring bias and premature closure, both of which limited consideration of a broad differential diagnosis, manifested in their reasoning during clerkships.

“*I was just kind of anchoring on the fact that the overnight person was very concerned about TB, and he had the weight loss, he had the coughing up blood and all the things, and he was born overseas and didn’t come to the U.S. until later. Yeah, so I was just very fixated on that and I didn’t do as thorough of a history and physical as I should have.”*

Methods taught in the CR course for addressing cognitive bias included deliberately maintaining multiple diagnostic considerations, re-analyzing data, and continuously reassessing the differential diagnosis. Students described efforts to follow these recommendations in clerkships to support their diagnostic accuracy.

“*I saw a lot of patients with a suspected stroke. I asked a lot of questions that were not only related to stroke, but things related to other possibilities that may present similarly.”*

*“So there’s definitely times in different clinics where I'm like, ‘Could it be that this thing is what it really is, but it's been labeled as this [other thing] all these years?’*

*“I struggle a lot on clerkships with maintaining a very broad differential. . .that's a surprisingly hard thing to do.”*

Students recognized instances where implicit biases linked with systemic and structural racism, which were also discussed in the CR course, impacted patient care.

*“Whenever we had patients for OB/GYN that were using drugs or babies born to mothers who use drugs, there was always a tendency to kind of attribute any signs and symptoms to drug use instead of [other things]. . .And you know we do have that history, so that is contributing to our diagnostic thinking, but I think that was definitely an area that I saw ripe for huge diagnostic error and kind of misattributing everything to, ‘Oh, because their mom is. . .on heroin or something,’ which is really sad. [Interviewer: What do you think were some of the underlying causes for that potential diagnostic error?] I think that is partially due to you know, systemic racism, structural racism, and implicit and explicit bias, which we learn a lot about, [but] I definitely didn’t expect it to be so inherent. I’m just kind of seeing different differential treatment of patients due to their race, even when they had both been in the same situation.”*

### Theme 2: CR Concept Reinforcers

Contextual factors may have influenced when and how students utilized CR concepts. For example, students were able to draw connections between contexts, such as specific specialties and practice settings, and their utilization of CR concepts in the workplace.

“*I feel that…all of the clinical reasoning skills that I have used throughout my medicine [clerkship] were entirely based on the foundation that was built in DR Block [pre-clinical CR course]. And I would not have survived or not have thrived the way I have without that foundation.”*

Students recognized that the approach to differential diagnosis was different depending on the context of a clerkship. For example, a student reflected on experiences during stroke call on the neurology rotation, during which there was frequent interaction with emergency department (ED) providers. The student noted that in the ED, the emphasis on “can’t miss” diagnosis is greater given the large volume of high acuity patients. These experiences enabled them to thoughtfully compare various clerkship contexts.

*“…in the ED, because they have a very different way of approaching patients [compared to neurology] where rather than ‘most likely,’ they’re most concerned about most deadly.”*

Although students made connections between reasoning concepts and specific clinical contexts, our data did not indicate whether different contexts prompted concept recognition or skill utilization.

Some attending physicians utilized CR concepts in the clinical setting, which reinforced the CR framework for students.

*“And that patient I saw in the ED with the seizure, or the syncope, that was a really good experience for me working with the attending, like hearing what her schema was, and just starting with ‘it’s seizure or syncope,’ and here are some big flags that can point you in one direction or the other, and then you can work farther down the differential for either of those.”*

Students reflected that input from their supervisors could help them to recognize areas for improvement in their reasoning. Interactions with some supervisors prompted students to be metacognitive about their reasoning (e.g., noting their cognitive tendencies), which was a core objective of the CR course.

*“Like it was really jumping out to me, but then the attending…did a couple of more things and she ultimately thought it was costochondritis. I had attached myself to her taking ibuprofen a couple of days ago or something like that and something else that made me think the more likely thing was peptic ulcer and I had to be…pulled back from that and think of the costochondritis. I think it is very easy for me to automatically just do what I think is most likely. And it’s harder for me ... I have to pull back and think about things that are less likely and not automatic by pulling apart the symptoms.”*

### Theme 3: CR Concept Diminishers

Contextual factors in the clerkship environment also created barriers that limited students’ application of CR concepts or the ability to discuss their reasoning. For example, time constraints sometimes led to briefer and more focused discussions that prevented students from asking questions about diagnostic reasoning.

*“I wanted to ask that [diagnostic] question, but I didn’t get the opportunity to because clinic was going way too fast that day.”*

*“Most of the time I walk out and in two minutes the preceptor is there [saying], ‘Oh okay, so let’s hear about so and so.’ I’m like, ‘I’m not ready at all.’”*

Occasionally supervising physicians instructed students to limit or constrain use of certain CR practices, while others expressed unfamiliarity with CR concepts, impacting students’ ability to practice or get feedback on their reasoning.

“*She [supervising physician] told me ‘Oh, there's no reason to write down the “can't miss” diagnoses. You can tell me that verbally, but don't write it down and know it has no place in the EMR.’”*

*“The ones that weren't as familiar with the reasoning, in terms of going through the differential, and explaining why each one was more, versus less, versus not likely, some attendings would say, ‘Oh, I'm old-school, I'm not familiar with that.’”*

## DISCUSSION

Students’ reflections on their reasoning highlighted factors that sustained, reinforced, or diminished use of the CR framework taught in a CR course. Contexts that aligned with the diagnosis-focused CR framework (e.g., internal medicine) and supervisors who were familiar with, modeled, or gave feedback relevant to CR concepts reinforced the CR framework. Supervisors who were less familiar with the CR framework or found it unhelpful tended to limit its use by students. Time constraints—felt by students and imposed by supervisors or by the pace of the clinical environment—limited students’ ability to either thoroughly conduct or communicate all the reasoning steps they intended to enact to diagnose a patient’s clinical problem.

We found that clerkship students used the CR framework taught in a pre-clinical CR course to describe their thought processes and practices when considering patients’ diagnoses. They described employing an intentional approach before (i.e., when preparing to see patients), during, and after (i.e., while writing notes and giving oral presentations) clinical encounters that was structured using core reasoning concepts. Students also recognized influences on their reasoning process, such as cognitive and implicit biases, and the impact of communication with patients and interprofessional colleagues. They reflected on the utility of CR principles in supporting efforts to minimize diagnostic error from premature closure or anchoring, overlooking diagnostic categories, or missing a diagnosis due to personal knowledge limits.

**Figure 1:**
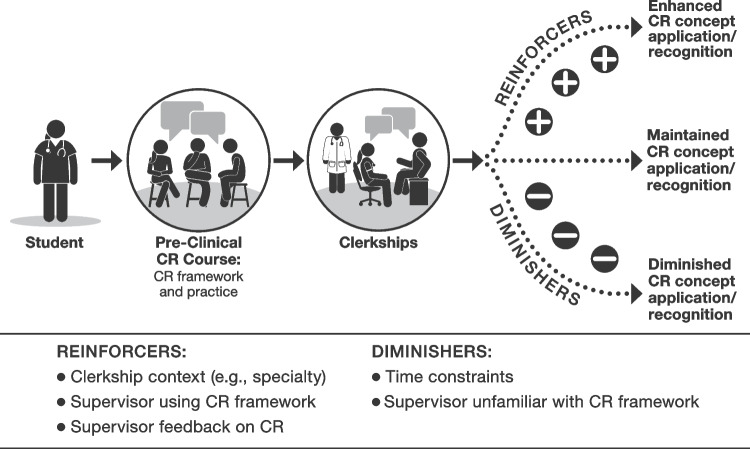
Reinforcers and diminishers of the use of clinical reasoning concepts from a pre-clinical reasoning course during clerkships.

Figure 1 depicts a model derived from our findings describing students’ application and recognition of reasoning concepts from a pre-clinical CR course in the clerkship setting, along with reinforcers and diminishers to their use. By reflecting on this blueprint, educators can take steps to reinforce and extend CR skills in clerkships. First, educational leaders can enhance faculty development to ensure more effective CR instruction. Fulton et al. noted that even clinical educators who are facile with basic science knowledge may not know how to support students’ learning in clerkships by making links between basic science and clinical care.^14^ Similarly, clinicians who feel confident in their CR capabilities may lack the skills needed to connect pre-clinical CR course terminology and principles with students’ clinical efforts in clerkships. Faculty development for CR teaching should create a shared mental model of the CR process and a common vocabulary to describe CR components.^23^ This recommendation aligns with students’ comments in our study about the educational value of having supervisors who explicitly discussed the reasoning framework.

Our findings also suggest that CR course directors should include instruction on how CR is conducted in different specialties and with interprofessional colleagues, and need to prepare students to use the CR framework for thinking and communicating even in time-limited situations. For instance, our CR course leaders are now integrating CR concepts that are germane to more procedural fields to prepare students to recognize and employ CR across the full range of clerkships. Clerkship didactics are another opportunity to reinforce CR concepts that may not be discussed frequently in the clinical learning environment.^12^ Reasoning concepts that were taught in the CR course but did not surface in students’ reflections (e.g., Bayesian reasoning) could be emphasized in clerkship didactics. Alternatively, CR concepts that never surface in the clinical years may be a signal to course directors that those topics deserve less attention in the pre-clinical phase. Discussions between pre-clinical and clinical educators could identify instances in each phase of education that could reinforce or refine the reasoning framework and related concepts.

Our study aligns with social learning theory, which predicts that the persistence of pre-clinical concepts depends on the interaction of students, supervisors, and clinical workplace practices.^24^ These connections have been seen in other areas of medical education. Laksov et al. interviewed anatomy and surgery teachers about the challenges that students have utilizing knowledge from anatomy courses in the clinical environment.^25^ Some teachers had strategies to address the problem, for example by emphasizing re-learning and repeating pre-clinical anatomical knowledge during surgery rotations.^25^ Fulton et al. studied senior medical students’ perceptions about facilitators and barriers to applying biochemical mechanisms to inform patient care.^14^ Drawing on social cognitive theory,^26^ they concluded that transfer of classroom knowledge to the clinical setting, which is often viewed as a cognitive issue (recalling information from memory), is also a sociocognitive phenomenon (a learning process influenced by reciprocal interactions between personal, social, and environmental factors). Other studies on transfer of learning from the classroom to the workplace similarly attribute effective transfer to characteristics of the student, the classroom, and the clinical workplace.^27^ Future studies could explore students’ internal motivations, self-efficacy, and strategies for employing and strengthening CR skills in clerkships.

Ecological psychology (EP) is another useful social learning theory that supports our model. EP emphasizes how learning is influenced by the environment and spotlights effectivities, which are the actions an individual is able to perform in a specific context, and affordances, which are the conditions necessary for the agent to perform those actions.^28^ In the language of EP, students develop effectivities for performing different steps in the CR framework through their pre-clinical education (including the CR curriculum), and the clerkship environment provides variable affordances for students to engage with the CR framework. The CR concept reinforcers (e.g., supervisors who modeled a schema) were positive affordances, while the CR concept diminishers (e.g., time constraints) were negative affordances. “Positive” and “negative” do not constitute value judgements, but rather reflect the degree to which the affordances supported or hindered the curricular goal of CR concept exposure or practice beyond the pre-clinical years. Future studies of CR in clerkships may benefit from employing EP as a grounding theory.

This study does not establish causality. Our data does not indicate whether the CR course was responsible for students’ understanding of CR concepts, which they may have come to understand through alternative experiences. Also, teachers who did not utilize the CR framework may still have imparted relevant reasoning skills through other mechanisms, e.g., providing meaningful feedback on students’ presentations.

A limitation of our study is that retrospective interviews were used to gather data. Direct observations of students and supervisors in the clinical environment may have yielded different insights, including evidence of CR skill development. Focus groups, which support more dynamic, synergistic discussions, could have revealed different insights, and could be a useful approach in future studies. Our study enrolled students who had completed clerkships emphasizing diagnostic reasoning. Students starting with clerkships where management (rather than diagnosis) was prioritized or where discussion of reasoning was limited by specialty culture may have revealed different perspectives on the scope of CR course concepts and practices that students noticed or learned. Our pre-clinical CR course focused on the individual clinician’s cognitive processes and was led by faculty trained in specialties that emphasize diagnostic reasoning. Future studies could include students who had completed a broader range of both diagnostic- and management-focused clerkships. A pre-clinical reasoning course that more strongly emphasizes systems, teams, or management concepts may influence students differently once they reach clerkships.

We found that students continued to use many reasoning concepts taught in a pre-clinical reasoning course during clerkships, and that contextual and supervisor factors could either facilitate or diminish students’ use of the CR framework. CR course directors can help students develop strategies to utilize reasoning concepts even in the face of time-pressure and can work with clerkship directors and faculty development leaders to find opportunities for reinforcement, extension, and coaching of reasoning concepts in the clinical environment.

## Supplementary Information

Below is the link to the electronic supplementary material.

Supplementary file1 (DOCX 20 KB)

## Data Availability

Restrictions apply to the availability of the data that support the findings of this study as interview transcripts were restricted to use by the study team by the Institutional Review Board (IRB) process and are not publicly available. The anonymized data can, however, be made available from the authors upon reasonable request and with the permission of the UCSF IRB.

## References

[1] **Balogh EP, Miller BT, Ball JR, eds.** The National Academies of Sciences, Engineering, and Medicine. *Improving Diagnosis in Health Care*. Washington, DC: National Academies Press; 2015. 26803862

[2] **Cooper N, Bartlett M, Gay S, et al.** Consensus statement on the content of clinical reasoning curricula in undergraduate medical education. *Medical Teacher*. 2021;43(2):152-159. 33205693 10.1080/0142159X.2020.1842343

[3] **Elizondo‐Omaña RE, Morales‐Gómez JA, Morquecho‐Espinoza O, et al.** Teaching skills to promote clinical reasoning in early basic science courses. *Anatomical Sciences Ed*. 2010;3(5):267-271. 10.1002/ase.178 20809498

[4] **Jacobson K, Fisher DL, Hoffman K, Tsoulas KD.** Integrated Cases Section: A Course Designed to Promote Clinical Reasoning in Year 2 Medical Students. *Teaching and Learning in Medicine*. 2010;22(4):312-316. 20936581 10.1080/10401334.2010.512835

[5] **Harendza S, Krenz I, Klinge A, Wendt U, Janneck M.** Implementation of a Clinical Reasoning Course in the Internal Medicine trimester of the final year of undergraduate medical training and its effect on students’ case presentation and differential diagnostic skills. *GMS Journal for Medical Education;* 2017; 34(5):Doc66. 29226234 10.3205/zma001143 PMC5704605

[6] **Keemink Y, Custers EJFM, Van Dijk S, ten Cate O.** Illness script development in pre-clinical education through case-based clinical reasoning training. *Int J Med Educ*. 2018;9:35-41. 29428911 10.5116/ijme.5a5b.24a9 PMC5834830

[7] **ten Cate O, Custers EJFM, Durning SJ, eds.** *Principles and Practice of Case-Based Clinical Reasoning Education: A Method for Preclinical Students*. Cham, Switzerland: Springer; 2018. 31314234

[8] **Waliany S, Caceres W, Merrell SB, Thadaney S, Johnstone N, Osterberg L.** Preclinical curriculum of prospective case-based teaching with faculty- and student-blinded approach. *BMC Med Educ*. 2019;19(1):31. 30674302 10.1186/s12909-019-1453-x PMC6343267

[9] **Moghadami M, Amini M, Moghadami M, Dalal B, Charlin B.** Teaching clinical reasoning to undergraduate medical students by illness script method: a randomized controlled trial. *BMC Med Educ*. 2021;21(1):87. 33531017 10.1186/s12909-021-02522-0 PMC7856771

[10] **Choi JJ, Gribben J, Lin M, Abramson EL, Aizer J.** Using an experiential learning model to teach clinical reasoning theory and cognitive bias: an evaluation of a first-year medical student curriculum. *Medical Education Online*. 2023;28(1):2153782. 36454201 10.1080/10872981.2022.2153782 PMC9718553

[11] **Rencic J, Trowbridge RL, Fagan M, Szauter K, Durning S.** Clinical Reasoning Education at US Medical Schools: Results from a National Survey of Internal Medicine Clerkship Directors. J Gen Intern Med. 2017;32(11):1242-1246. 28840454 10.1007/s11606-017-4159-y PMC5653563

[12] **Duca NS, Glod S.** Bridging the Gap Between the Classroom and the Clerkship: A Clinical Reasoning Curriculum for Third-Year Medical Students. *MedEdPORTAL*. Published online January 25, 2019:10800. 10.15766/mep_2374-8265.10800 PMC6507921 31139730

[13] **Castillo JM, Park YS, Harris I, et al.** A critical narrative review of transfer of basic science knowledge in health professions education. *Med Educ*. 2018;52(6):592-604. 29417600 10.1111/medu.13519

[14] **Fulton TB, Collins S, Van Der Schaaf M, O’Brien BC.** Connecting Biochemistry Knowledge to Patient Care in the Clinical Workplace: Senior Medical Students’ Perceptions about Facilitators and Barriers. *Teaching and Learning in Medicine*. 2023;35(4):398-410. 35796605 10.1080/10401334.2022.2084400

[15] **Connor DM, Narayana S, Dhaliwal G.** A clinical reasoning curriculum for medical students: an interim analysis. *Diagnosis*. 2022;9(2):265-273. 10.1515/dx-2021-0112 34904425

[16] **Connor DM, Dhaliwal G, Bowen JL.** Teaching Clinical Reasoning in Medical Education Courses (Chapter). In: Higgs J, Jensen GM, Loftus S, Christensen N, eds. *Clinical Reasoning in the Health Professions, Fourth Edition*. Amsterdam, Netherlands: Elsevier; 2019:345-358.

[17] **Ryan MS, Lee B, Richards A, et al.** Evaluating the Reliability and Validity Evidence of the RIME (Reporter-Interpreter-Manager-Educator) Framework for Summative Assessments Across Clerkships. *Acad Med*. 2021;96(2):256-262. 33116058 10.1097/ACM.0000000000003811

[18] **Charmaz, K.** *Constructionism and the Grounded Theory Method; Handbook of Constructionist Research*. Vol 1. 1st ed. New York, NY: The Guilford Press; 2008.

[19] **Watling CJ, Lingard L.** Grounded theory in medical education research: AMEE Guide No. 70. *Medical Teacher*. 2012;34(10):850-861. 22913519 10.3109/0142159X.2012.704439

[20] **Charmaz K.** The Power of Constructivist Grounded Theory for Critical Inquiry. *Qualitative Inquiry*. 2017;23(1):34-45.

[21] The Clinical Problem Solvers. Available at: https://clinicalproblemsolving.com/. Accessed November 9, 2024.

[22] **Saposnik G, Redelmeier D, Ruff CC, Tobler PN.** Cognitive biases associated with medical decisions: a systematic review. *BMC Med Inform Decis Mak*. 2016;16(1):138. 27809908 10.1186/s12911-016-0377-1 PMC5093937

[23] **Maciuba JM, Mallory R, Surry L, et al.** Teaching Students How to Think: A Longitudinal Qualitative Study of Preclerkship Clinical Reasoning Instruction. *Military Medicine*. 2023;188(Supplement_2):50-55. 37201489 10.1093/milmed/usad036

[24] **Mukhalalati B, Elshami S, Eljaam M, Hussain FN, Bishawi AH.** Applications of social theories of learning in health professions education programs: A scoping review. *Front Med (Lausanne)*. 2022;9:912751. 35966845 10.3389/fmed.2022.912751 PMC9367215

[25] **Bolander Laksov K, Lonka K, Josephson A.** How do medical teachers address the problem of transfer? *Adv in Health Sci Educ*. 2008;13(3):345-360. 10.1007/s10459-006-9048-9 17203269

[26] **Schunk DH, ed.** Social cognitive theory. In *Learning Theories: An Educational Perspective*. 8th ed. New York, NY: Pearson; 2020.

[27] **Peters S, Clarebout G, Van Nuland M, Aertgeerts B, Roex A.** A Qualitative Exploration of Multiple Perspectives on Transfer of Learning Between Classroom and Clinical Workplace. *Teaching and Learning in Medicine*. 2018;30(1):22-32. 28753068 10.1080/10401334.2017.1339605

[28] **Watsjold BK, Ilgen JS, Regehr G.** An Ecological Account of Clinical Reasoning. *Acad Med*. 2022;97(11S):S80-S86. 35947479 10.1097/ACM.0000000000004899

