# Supplementary material for: Clerkship Students’ Use of Clinical Reasoning Concepts After a Pre-clinical Reasoning Course
Source: J Gen Intern Med. 2025 Jan 2;40(6):1359–66. doi: 10.1007/s11606-024-09279-4 (PMC12045889; doi:10.1007/s11606-024-09279-4)
Supplement: Supplementary file 1 — Supplementary file1 (DOCX 20 KB) [file 11606_2024_9279_MOESM1_ESM.docx]

**Appendix A: Clinical Reasoning Course Components (Content and Teaching Approaches)**

| Component | Session Content | Teaching Approaches |
| --- | --- | --- |
| Foundational Reasoning Concept Workshops | 4 half-day day reasoning workshops focused on core clinical syndromes (chest pain, transient loss of consciousness, the red leg, vertigo) integrated with simultaneous content in core basic science courses and highlighting foundational components of the CR framework (problem representation, illness scripts, diagnostic schema) | Interactive small group sessions facilitated by faculty coaches in longitudinal clinical skills component of curriculum emphasizing compare/contrasting of overlapping diagnoses for common clinical problems  Scaffolding includes completion of partially filled-in problem representations, illness scripts, schema |
| Interactive, Facilitated Small Groups | 7 case-based small groups covering common clinical syndromes (abdominal pain in a toddler, respiratory distress in an infant, dyspnea in an older adult, weakness altered mental status, hypotension in an older adult, abdominal pain in pregnancy)  1 case-based session on considering goals of care during diagnostic process  1 case-based session on ethical dilemmas during the diagnostic process  1 case-based session on communicating clinical reasoning during oral presentations  1 case-based session on Bayesian reasoning in the diagnostic process | Interactive small group sessions facilitated by clinical faculty (primarily from internal medicine, family medicine, emergency medicine, pediatrics, and neurology) with specific feedback on students’ diagnostic reasoning in the Assessment and Plan  Scaffolding includes completion of partially filled-in compare/contrast illness script grids  Several sessions feature videos portraying clinician-patient encounters and live standardized patient interviews to model and facilitate practice with diagnostic communication |
| Large Group Sessions | 2 case-based sessions with expert clinicians narrating their diagnostic approach  1 case-based session on diagnostic error with a focus on interprofessional collaboration on diagnosis  1 session on implicit bias in diagnosis  1 session on diagnostic error with patient and faculty panel | Use patient and clinician narratives to model reasoning, consider risks for and impact of diagnostic error, and to discuss systems- and individual strategies for managing diagnostic error |
| Integration with Other Courses | Intentional connections with clinical skills curriculum (emphasis on hypothesis-driven physical exam and reflections after clinical preceptorships); clinical syndromes in reasoning sessions are timed to connect with content being taught in parallel basic science courses; connection with longitudinal interprofessional curriculum and systems science/quality improvement curriculum during the CR course | Utilize a common set of CR framework concepts and terms in linked parts of the curriculum to reinforce CR knowledge and demonstrate connections with other learning throughout the pre-clinical curriculum across the domains of clinical skills, medical knowledge, interprofessional teamwork, and systems science |

**Appendix B: Interview Guide**

**Structured Interview Script**

**Opening:**

Thank you for sharing your experiences and perspective with us today. Before we start, I want to mention that there are no ‘right answers’ to these questions—we are very interested in your experiences with reasoning in clerkships and encourage you to share both times that things went well, and times that were more challenging. I will be recording our conversation, but your responses will be transcribed and de-identified—you will remain anonymous, and your name will **not** be shared with others when we analyze and share our data.

*[If students brought a patient note with them]:* Before we start the interview, please spend a couple of minutes reviewing your patient note. You can use this patient encounter as an example during our interview if that’s helpful. Please also draw on experiences with other patients during your clerkship(s) when answering questions below. Please do not share any protected health information (e.g., names or medical record numbers) in your answers.

**Metacognitive Domain:**

****Notes about sensitizing concepts that informed development of interview questions are included below in italics. These sensitizing concepts were not shared or discussed during the interview itself, nor were they shared with students serving as interviewers. Rather, these concepts were utilized by the research design team when drafting and revising the structured interview guide. Questions themselves were intended to be open-ended to avoid leading students to specific responses.***

1. Tell me about an inpatient or outpatient you saw with a new chief concern where you spent significant time thinking through their diagnosis. This can be the patient whose note you reviewed just now, or a different patient from your clerkships.
2. What made this case a diagnostic challenge for you?
3. *Besides* looking up the patient’s history in their chart, after hearing the patient’s chief concern, how do you generally organize your thinking to see a patient for the first time? [Before you enter the room] *[Sensitizing Concepts: Querying potential hypothesis-driven nature of preparation process and use of reasoning frameworks such as diagnostic schema to structure preparation]*
   1. [If specifics not given yet, ask: Can you give a specific example?]
4. What is going on in your mind *as you are performing* an initial H&P for a patient presenting with a new clinical problem? To describe this process, you can use the same patient discussed before, or another patient where you were trying to develop your differential diagnosis.[While you are doing the H&P] *[Sensitizing Concepts: Querying potential hypothesis-driven nature of approach to H&P and use of reasoning frameworks such as problem representation and diagnostic schema to structure H&P]*
5. *Besides* accessing references (e.g. UpToDate), after your encounter with a new patient, what *cognitive processes or strategies* do you use to develop and refine your differential diagnosis? [After you’ve left the room] *[Sensitizing Concepts: Querying potential use of reasoning frameworks such as problem representation, diagnostic schema, and justified, prioritized differential diagnosis in differential diagnosis development]*
   1. [Interviewer note: Encourage specifics for the same, or a different patient]
6. What framework or process do you use to communicate your reasoning to your team or attending during your oral presentation (for the same, or another patient where you were trying to develop your differential diagnosis)? *[Sensitizing Concepts: Querying potential use of reasoning frameworks such as problem representation, diagnostic schema, and justified, prioritized differential diagnosis in communicating about reasoning]*
   1. [If no specifics given, can ask: are there places in your note where you see evidence of how you were applying your reasoning approach to this patient’s case that would have helped you to communicate your reasoning to your team?]
7. Were there incidents where your resident/fellow/attending advised you to approach diagnostic thinking differently than how you had been taught to do so prior to clerkships? [If yes, please describe.]
8. Moving beyond the patient(s) we have discussed so far, describe a situation during your clerkship when you noticed the potential risk for diagnostic error in your own thinking, or in the thinking of your clinical team (e.g. during rounds)? *[Sensitizing Concepts: Querying potential integration of learning about cognitive and other biases, as well as issues with interprofessional team and/or patient communication in contributing to the risk for diagnostic error]*
   1. [If no examples given, ask: What about a potential error that was avoided?]
   2. [If not already answered, ask: What do you think were some underlying causes for this risk for diagnostic error?]
   3. [If not already answered, ask: How did you and/or your team respond to the risk for diagnostic error in general?]

**Reflection on Clinical Reasoning Curriculum (pending time):**

1. Did interns/residents/fellows/attendings you’ve worked with so far have an understanding of the diagnostic framework/language you learned in the pre-clerkship curriculum? [If no, did that lead to any problems?]
